# P-477. Healthcare-Associated Infections among Infants in Tennessee, 2015–2023

**DOI:** 10.1093/ofid/ofaf695.692

**Published:** 2026-01-11

**Authors:** Jordan Morris, Marissa M Turner, Vicky Lindsey, Christopher D Evans, Christopher Wilson, Ashley Gambrell

**Affiliations:** Tennessee Department of Health, Nashville, TN; Tennessee Department of Health, Nashville, TN; Tennessee Department of Health, Nashville, TN; Tennessee Department of Health, Nashville, TN; Tennessee Department of Health, Nashville, TN; Tennessee Department of Health, Nashville, TN

## Abstract

**Background:**

Infant patients are a vulnerable group, in part due to healthcare-associated infections (HAI) which occur while in a healthcare facility from medical devices, surgical procedures, or other nosocomial transmission. While the United States has a wealth of literature on the prevalence of healthcare-associated infections in Neonatal and Pediatric Intensive Care Units, Tennessee lacks such studies specifically. The National Healthcare Safety Network (NHSN) stores data on the most common HAIs among infants. We examined Tennessee data to identify the frequency of HAIs among infants by HAI type from 2015–2023 and describe the demographics of infants infected with HAIs in Tennessee.

**Figure:**
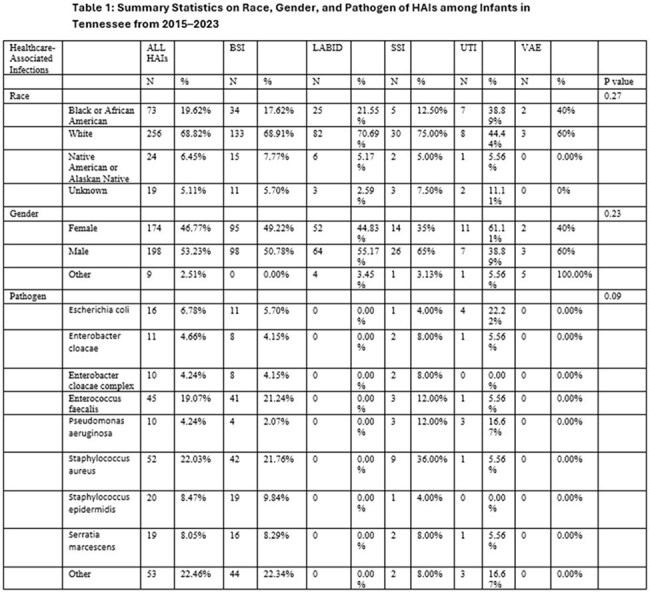

**Figure:**
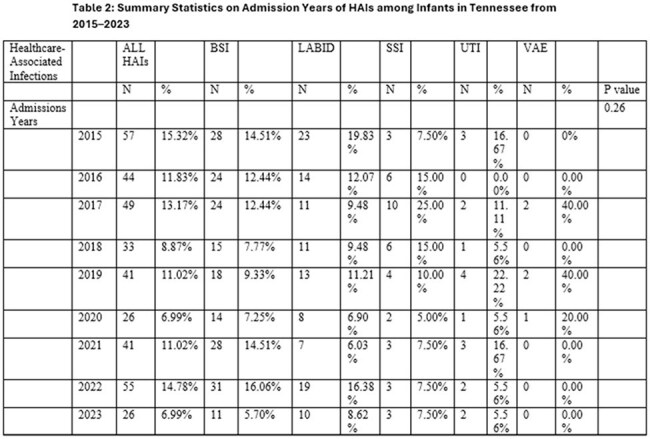

**Methods:**

Data were retrieved from NHSN, and cases were selected from infants in Tennessee Acute Care Hospitals, aged one year or younger at the time of infection. Data were matched with the Tennessee Hospital Discharge Data Set (HDDS) to ensure more robust demographic data, and subsequent analyses were completed assessing distributions of HAIs and patient characteristics, using SAS v9.4.

**Figure:**
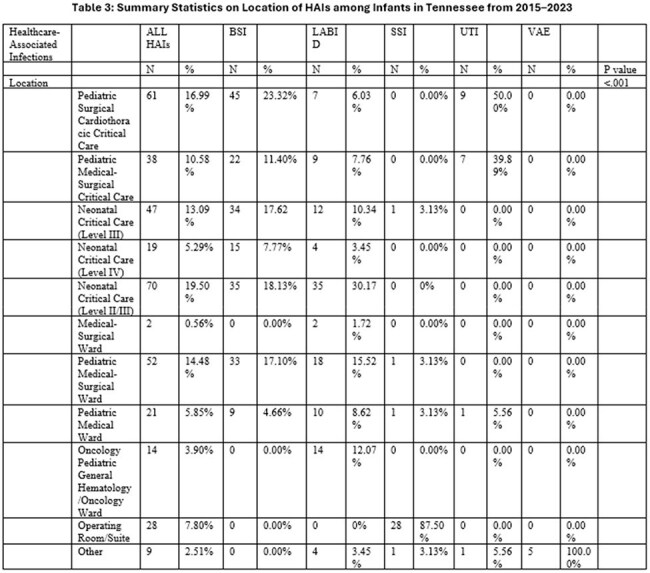

**Results:**

There were 372 HAIs identified in infants during the study, as shown in the tables. Central line-associated bloodstream infections were the most common, accounting for 193 (56.45%) cases. LabID events, including Methicillin-resistant *S. aureus* bacteremia and *C. difficile* infections accounted for 116 (31.18%) cases. Annually, 2015 reported the highest incidence of HAIs at 57 (15.32%) cases, while 2020 reported the lowest at 26 (6.99%). The most HAIs occurred in Level II/III Critical Care areas at 70 (18.82%) cases, and Pediatric Surgical Cardiothoracic Critical Care at 61 (16.40%). The leading pathogens identified were *S. aureus*, contributing to 52 (13.97%) cases and *E. faecalis* with 45 (12.09%) cases.

**Conclusion:**

There was significant variation among patient characteristics by HAI type and the number of HAIs annually. Although declining from 2015 to 2020, HAI rates in infants rose slightly again after 2020,

highlighting the need for continued infection prevention efforts and targeted interventions. While this variation is possibly due to limited sample size, further research should evaluate factors significantly associated with HAI acquisition in infants and assess if these factors occur equally across the state.

**Disclosures:**

All Authors: No reported disclosures

